# Management of Mandibular Condyle Fractures in Pediatric Patients: A Multicentric Retrospective Study with 180 Children and Adolescents

**DOI:** 10.3390/jcm13185455

**Published:** 2024-09-14

**Authors:** Gian Battista Bottini, Wolfgang Hitzl, Maximilian Götzinger, Constantinus Politis, Kathia Dubron, Mario Kordić, Anamaria Sivrić, Petia Pechalova, Angel Sapundzhiev, Valfrido Antonio Pereira-Filho, Luis Fernando de Oliveira Gorla, Emil Dediol, Boris Kos, Tabishur Rahman, Sajjad Abdur Rahman, Sahand Samieirad, Timothy Aladelusi, Vitomir S. Konstantinovic, Marko Lazić, Aleš Vesnaver, Anže Birk, Karpal Singh Sohal, Sean Laverick, Euan Rae, Maria Beatrice Rossi, Fabio Roccia, Federica Sobrero

**Affiliations:** 1 Department of Oral and Maxillofacial Surgery and Center for Reconstructive Surgery, University Hospital of the Private Medical University Paracelsus, 5020 Salzburg, Austria; ma.goetzinger@salk.at; 2 Research and Innovation Management, Biostatistics, Department of Ophthalmology and Optometry, Research Program Experimental Ophthalmology and Glaucoma Research, Paracelsus Medical University, 5020 Salzburg, Austria; wolfgang.hitzl@pmu.ac.at; 3 Department of Oral and Maxillofacial Surgery, University Hospitals Leuven, Kapucijnenvoer 7, 3000 Lueven, Belgium; constantinus.politis@kuleuven.be (C.P.);; 4 Clinic for ENT and OMS, University Clinical Hospital, 88000 Mostar, Bosnia and Herzegovina; korda333@gmail.com (M.K.); sivrica@yahoo.com (A.S.); 5 Department of Oral Surgery, Faculty of Dental medicine, Medical University of Plovdiv, 4000 Plovdiv, Bulgaria; pechalova@abv.bg (P.P.); angelsapundjiev@abv.bg (A.S.); 6 Department of Diagnosis and Surgery, Division of Oral and Maxillofacial Surgery, São Paulo State University, UNESP, Araraquara 14801903, SP, Brazil; valfrido.pereira-filho@unesp.br (V.A.P.-F.); fernando.gorla@gmail.com (L.F.d.O.G.); 7 Department of Maxillofacial Surgery, University Hospital Dubrava, 10000 Zagreb, Croatia; emildediol@yahoo.com (E.D.); kosboris7@gmail.com (B.K.); 8 Department of Oral and Maxillofacial Surgery, Aligarh Muslim University, Aligarh 202002, India; tabishalig05@gmail.com (T.R.); sajjadar1979@gmail.com (S.A.R.); 9 Oral & Maxillofacial Surgery Department, Mashhad Dental School, Mashhad University of Medical Sciences, Mashhad 91779-48564, Iran; samieerads@mums.ac.ir; 10 Department of Oral and Maxillofacial Surgery, College of Medicine, University of Ibadan, Ibadan 200005, Nigeria; drtimmylee@gmail.com; 11 Clinic of Maxillofacial Surgery, School of Dentistry, University of Belgrade, 11000 Belgrade, Serbia; v.konstantinovic@stomf.bg.ac.rs (V.S.K.); marko.lazic@stomf.bg.ac.rs (M.L.); 12 Department of Maxillofacial and Oral Surgery, University Medical Centre, 1000 Ljubljana, Slovenia; ales.vesnaver@gmail.com (A.V.);; 13 Department of Oral and Maxillofacial Surgery, Muhimbili University of Health and Allied Sciences, Dar es Salaam 65001, Tanzania; karpal@live.com; 14 Department of Oral and Maxillofacial Surgery, University of Dundee, Dundee DD1 4HR, UK; sean.laverick2@nhs.scot (S.L.); euan.rae1@nhs.scot (E.R.); 15 Division of Maxillofacial Surgery, Città della Salute e della Scienza Hospital, University of Turin, 10126 Turin, Italy; mariabeatrice.rossi@unito.it (M.B.R.); fabio.roccia@libero.it (F.R.); federica.sobrero@unito.it (F.S.)

**Keywords:** mandibular condyle fractures, pediatric mandibular condyle fractures, pediatric maxillofacial trauma, expectant management, maxillomandibular fixation, open reduction and internal fixation

## Abstract

**Background**: Mandibular condyle fractures in pediatric patients can lead to crippling sequelae such as ankylosis, pain and facial deformity if not managed properly. However, there is no consensus on the best approach for treating these fractures in children. **Objective**: This study aimed to describe the management of mandibular condyle fractures in growing patients across 14 maxillofacial departments worldwide. **Methods**: A retrospective multicenter study was conducted on children and adolescents aged 0 to 16 who had at least one mandibular condyle fracture. This study included patients who underwent expectant, closed, or open management and were treated over an 11-year period. **Results**: 180 patients had at least one mandibular condyle fracture, and 37 had a second condylar fracture. One hundred sixteen patients (65%) were males, and 64 (35%) were females (ratio 1.8:1). An expectant strategy was chosen in 51 (28%) patients, a closed treatment—stand-alone maxillomandibular fixation (MMF)—in 47 (26%), and open reduction and internal fixation (ORIF) was performed in 82 (46%) patients. The management varied significantly between the different departments (*p* < 0.0001). Significant differences were also identified between the fracture type (non-displaced, displaced or comminuted) and the management of the 180 patients with a single condylar fracture. Out of 50 non-displaced fractures, only 3 (6%) had ORIF, 25 (50%) had expectant management, and 22 (44%) had MMF. Out of 129 displaced fractures, 79 (62%) had ORIF, 25 (19%) had a soft diet, and 25 (19%) had MMF. **Conclusions**: Expectative management, MMF, and ORIF were all effective in treating pediatric mandibular condyle fractures, with a low incidence of complications and asymmetry.

## 1. Introduction

Pediatric maxillofacial fractures represent up to 15% of the total maxillofacial fractures in children and adults [1,2,3,4]. These fractures are rare in children under five years of age and are primarily due to falls. However, their incidence and causes vary in older children and adolescents across different publications and countries. Together with falls, road traffic accidents, sports and assaults are the most common etiologies [3,4,5,6]. Mandibular fractures, in turn, represent 20 to 50% of pediatric facial fractures, and the condylar process is commonly involved [2,3,5]. A recent multicentric prospective survey of pediatric maxillofacial trauma, including 322 patients with 474 fractures, reported 82 condylar fractures (17%) more than any other localization and was followed by the nose with 72 fractures [4]. Condylar fractures may easily escape diagnosis, especially in young children where cooperation and communication are limited, and computed tomograms (CT) can be labor-intensive due to the need for general anesthesia and frowned upon due to exposure to ionizing radiation. However, these injuries can have crippling sequelae, such as ankylosis and facial deformity in some patients, if not recognized and appropriately managed [5,7,8,9]. Because of their relevance, pediatric mandibular condyle fractures are a topic of great clinical interest. Several management strategies have been tried and described: expectative management (analgesia, soft food and follow-up), functional protocols with guiding elastics or with orthodontic appliances and exercises, maxillomandibular fixation (MMF), and open reduction and internal fixation (ORIF) [2,5,7,8,9]. Over the last two decades, there has been a shift towards open surgery in mainland Europe, following a similar trend in adult patients. The latter was based on the proven superiority of ORIF versus closed treatments, not only for fractures with severe displacement or dislocation but also for moderately displaced condylar fractures, defined as the deviation of 10° to 45° or shortening of the ascending ramus > 2 mm [10,11]. However, there is no consensus about the treatment of pediatric patients [3,7,8]. Regardless of the protocol, the goal is to restore pain-free temporomandibular joint function with a normal range of motion, pre-traumatic occlusion, facial symmetry, and expected growth. The World of Oral and Maxillofacial Trauma (WORMAT) project is a multicentric international collaboration of several maxillofacial units to study maxillofacial trauma. Fourteen centers from four continents took part in the present analysis on pediatric maxillofacial trauma, which are listed below in alphabetic order (Table 1).

The primary research question in this paper was to describe the authors’ management of pediatric mandibular condyle fractures and to assess whether there were differences in the outcomes between expectant management with a soft diet, closed treatment with MMF, and open surgery (ORIF) in the study sample. The null hypothesis being tested was that there were no significant differences in the outcome or number of complications between the above three types of management.

## 2. Patients and Methods

This retrospective multicentric study focused on children and adolescents aged 0 to 16 with complete clinical and radiological records admitted to the above departments from 1 January 2011 to 31 December 2021, having at least one mandibular condyle fracture that underwent either expectant, closed, or open management. The minimum follow-up was six months.

Exclusion criteria were the absence of condylar fractures, age older than 17, history of previous maxillofacial fractures, and craniofacial abnormalities. Additional non-condylar mandibular fractures (ramus, body, parasymphysis and symphysis) and craniofacial fractures were excluded by the present analysis and have been dealt with elsewhere or will be the topic of future articles of the WORMAT pediatric group [1].

Patients’ identities were only known to the unit researchers in which they were treated and were not shared with the other centers. Each patient received a patient identification number at the enrolling unit for data pseudonymization.

The following data were extracted from the clinical notes and the imaging: age, sex, cause of injury, fracture level (condylar head, -neck or -base according to Loukota and subsequent modification by Neff et al.) fracture type (non-displaced, displaced, and comminuted), associated mandibular and maxillofacial fractures and stage of dentition [12,13].

For schematic drawings and a detailed description of the latest modification of condylar fractures classification, which is adopted by the Arbeitsgemeinschaft für Osteosynthesenfragen (AO), the reader is referred to the excellent paper of Neff et al. [13]. Briefly, condylar head and neck fractures refer to the anatomical head and neck of the condylar process. The condylar base corresponds to a triangular ramus area between the sigmoid and masseteric notch lines [13]. The above two lines are perpendicular to the posterior ramus line, which is tangential to the lateral pole of the condylar head and the gonion [13]. The lower border of the condylar base is a line connecting the nadir of the sigmoid notch to the masseteric notch [13]. In the AOCMF classification, when a fracture crosses the neck and base border and at least 1/3 of the fracture length is above the sigmoid notch line, it is classified as a neck fracture [13]. Non-displaced fractures present as a fracture line within the condylar process, without alterations in its contours and complete apposition of the fragments; displaced fractures imply a discontinuity in the condylar process with loss of contact between the fragments.

According to the dentition stage, the patients were divided into three groups: deciduous (0–5 years), mixed (6–11 years), and permanent dentition (12–16 years). Furthermore, the time elapsed between the accident and the start of the intervention and the type of management were registered. The management was divided into three categories: expectant, closed or open.

Expectant management entailed soft food and analgesia as required; closed management consisted of maxillomandibular fixation or intermaxillary fixation (MMF or IMF), achieved through a variety of means such as arch bars, brackets, splints and self-tapping self-drilling screws, wires and elastics, according to the surgeon’s preference. Finally, open management involved ORIF, performed under general anesthesia.

No patient underwent functional protocols with bite-raising appliances and or elastic traction.

For the patients who underwent ORIF, the authors reported the type of hardware used for fracture fixation (titanium or resorbable), whether re-do operations had to be performed, whether MMF was used after ORIF, whether plates were removed later, and for what reason.

Furthermore, for all patients, regardless of their management, complications such as persistent pain and clicking of the temporomandibular joint (TMJ) facial asymmetry, reduced mouth opening, deviation on opening, malocclusion, infections and nerve injuries were documented.

The outcomes were considered ideal if the patients were well, occlusion restored to the pre-injury situation, with a maximal interincisal distance of at least 35 mm, without deviation or deflection from the midline on maximum opening, lateral excursions of at least 7 mm, a regular diet, absence of pain and clicking on mastication, absence of deformities and asymmetries on inspection and postoperative imaging, with the absence of nerve injuries and of growth anomalies.

Due to the retrospective, non-interventional nature of the study, 13 out of 14 participating departments did not require ethics committee approval. However, the Belgian unit obtained formal ethics approval (reference number S67588).

This study was conducted according to the principles of the Declaration of Helsinki and the identities of the patients were safeguarded [14].

Statistical methods: Data were checked for consistency. Pearson’s Chi-squared tests were used to analyze cross-tabulation tables with more than 4 cells. If assumptions for performing a classical Pearson Chi-Squared test were not met, *p*-values were computed by applying Monte Carlo methods with 4000 Monte Carlo samples. Pairwise comparisons and 2 × 2 tables were analyzed using Fisher’s Exact test. No adjustment for multiple testing was carried out. All reported tests were two-sided with a significance level of 5%. All statistical analyses in this report were performed using STATISTICA 13 (Hill, T. & Lewicki, P. Statistics: Methods and Applications. StatSoft, Tulsa, OK and Wolfram Research, Inc., Mathematica, Version 13., Champaign, IL, USA, 2022).

## 3. Results

### 3.1. Epidemiology

Of 424 pediatric patients with mandibular fractures managed in the above fourteen units over 11 years, 180 (42%) patients had at least one mandibular condyle fracture, and 37 (9%) patients had a second condylar fracture. One hundred sixteen patients (65%) were males and 64 (35%) were females (ratio 1.8:1). In total, 21 patients had deciduous dentition (0–5 years), 55 had mixed dentition (6–11 years), and 104 had permanent dentition (12–16 years).

**Figure 1:**
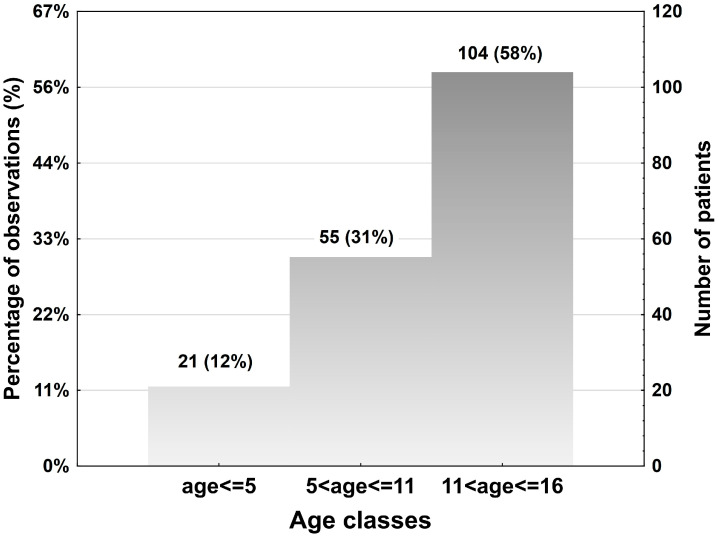
Age classes in the study sample.

A histogram with the three age classes in the study sample is shown below (Figure 1).

### 3.2. Etiology

The causes of injury were road traffic accidents (RTAs) in 63 (35%) patients, falls in 60 (33%), sports accidents in 42 (24%), assault in 10 patients (6%) and other non-specified accidents in 5 patients (2%). The statistical analysis showed significant differences between the younger children (0–5) and the second age group (6–11) as well as between the first (0–5) and the third (12–16) age groups concerning RTAs (*p*-value = 0.013 and 0.012 respectively), indicating a much higher prevalence of RTAs in the second and third age group. On the contrary, falls were significantly more prevalent in the first age group compared to the second and third (*p* = 0.0049 and 0.0001, respectively).

**Figure 2:**
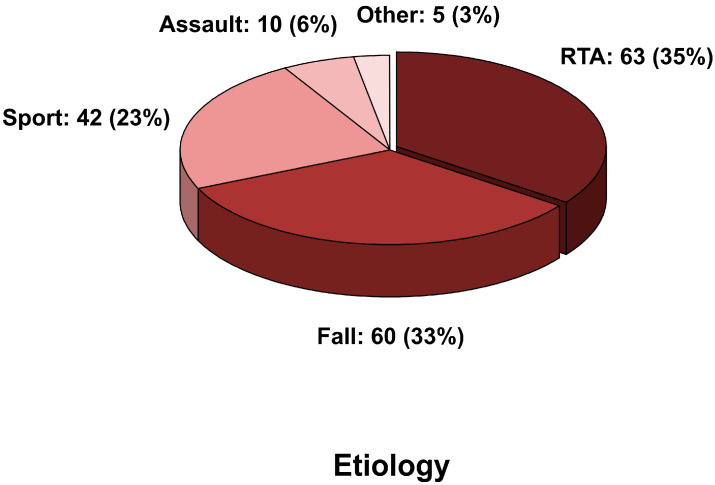
Pie chart representing the distribution of fracture causes.

A pie chart with the distribution of the fracture causes is shown below (Figure 2).

### 3.3. Fractures Localizations and Patterns

A total of 217 mandibular condyle fractures were localized as follows: 47 (22%) basis-, 92 (52%) neck- and 78 (36%) condylar head fractures. Overall, 35 of the 180 patients also had a third non-condylar mandibular fracture. The 180 patients had an additional 107 non-condylar mandibular fractures for a total of 324 mandibular fractures. One hundred sixty-eight patients had various patterns of mandibular fractures, with no other additional sites involved, whereas twelve patients had additional mid-facial fractures. Out of 180 patients with at least one condylar fracture, 70 (38%) had a second mandibular fracture (condylar or non-condylar); among them, 37 had a double condylar fracture. Finally, 37 patients (20%) had a third non-condylar mandibular fracture. The double and triple mandibular fractures displayed various combinations and no prevailing pattern.

Concerning the 180 patients with at least one condylar fracture and the 37 with a double condylar fracture, there was no significant association between the age groups and the fracture level (condylar base, -neck and -head) (*p* = 0.2 in both cases).

Similarly, there was no significant association between the fracture type (non-displaced, displaced or comminuted) and the three age groups (deciduous, mixed and permanent dentition) among the 180 patients with single condylar fracture (*p* = 0.05) and the 37 patients with a double condylar fracture (*p* = 0.69).

No significant association was found between the fracture level and the fracture type in the 180 patients with a single condyle fracture (*p* = 0.48) and the 37 patients with a double condyle fracture (*p* = 0.97).

### 3.4. Management Options: Expectative, Closed and ORIF

The time between the accident and treatment ranged from 1 to 3 days.

In total, 32 (18%) patients received antibiotics and corticosteroids for one week or longer, 137 (76%) patients received antibiotics for one week or longer, and 11 (6%) patients had neither antibiotics nor corticosteroids.

An expectant strategy was chosen in 51 (28%) patients, a closed treatment (stand-alone MMF) in 47 (26%), and ORIF was performed in 82 (46%) patients.

The management varied significantly between the different centers (*p* < 0.0001). Table 2 shows the number of patients in each unit and their treatment. In Ljubljana (Slovenia), Mashhad (Iran), São Paulo (Brazil) and Salzburg (Austria), the great majority underwent ORIF. In contrast, the surgeons in the ten remaining units preferred an expectative or closed strategy.

In the 82 ORIF patients, an extraoral approach was used for 70 patients and intraoral access for 12, one of which was performed with endoscopic assistance.

Fracture fixation was achieved with titanium plates or screws in 78 (95%) patients and with resorbable plates and screws in 4 (5%).

The osteosynthesis in condylar base and neck fractures was accomplished with two linear plates or a 3-D trapezoid plate. Two positional or lag screws were used in condylar head fractures as per expert recommendations [15].

Ninety-three patients (52%) had MMF, and eighty-seven (48%) did not. There was no significant association between the number of mandibular fractures (single, double or triple) and the use of MMF (*p* = 0.33). Of the 93 patients with MMF, 47 were treated with MMF as a stand-alone treatment (closed treatment), and the remaining 46 patients had MMF following ORIF. Therefore, in the ORIF group (82 patients), 36 (44%) had ORIF without postoperative MMF, whereas 46 patients (56%) had ORIF and MMF. MMF ranged from one to more than four weeks.

No significant difference was found in the type of management (expectant, closed or ORIF) among the three fracture localizations (condylar base, -neck or head) in the 180 patients with single condyle fracture (*p* = 0.09). However, with a *p*-value = 0.09, the association between condylar base fracture and ORIF was close to being significant, suggesting a tendency to treat single condylar base fractures with ORIF, with 24 (58%) open surgeries out of 41 condylar base fractures. In the group of 37 patients with double condylar fracture, no association was found between fracture level and management (*p* = 0.57).

Significant associations were identified between the fracture type (non-displaced, displaced or comminuted) and the management (expectant, closed or ORIF) of the 180 patients with a single condylar fracture. Out of 50 non-displaced fractures, only 3 (6%) had ORIF, 25 (50%) had expectant management, and 22 (44%) had MMF. Out of 129 displaced fractures, 79 (62%) had ORIF, 25 (19%) had a soft diet, and 25 (19%) had MMF. Finally, the remaining patient with a single comminuted fracture had an expectant management. Significant differences were observed between displaced (19%) and non-displaced fractures (44%) concerning MMF (*p* = 0.001). Furthermore, significant differences were seen in expectant management between displaced (19%) and non-displaced fractures (50%) (*p* < 0.0001). Finally, significant differences were also found about ORIF in displaced (62%) and non-displaced fractures (6%) (*p* < 0.0001).

In the group of 37 patients with a second condylar fracture, out of 14 non-displaced fractures, 10 (72%) had expectant management and 4 (28%) had MMF. Out of 21 displaced fractures, 7 (33%) had ORIF, 10 (48%) had a soft diet and follow-up and 4 (19%) had MMF. The remaining two comminuted fractures were treated with ORIF (1 patient) or soft diet (1 patient). In this group of 37 patients, a significant difference was seen between displaced (33%) and non-displaced fractures (0%) in relation to open treatment (*p* = 0.027).

There was no significant association between age groups and the management strategy in the 180 patients with a single condylar fracture (*p* = 0.45).

Of the 37 patients with double condylar fracture, 9 belonged to the deciduous dentition group, and all (100%) had expectant management. Overall, 13 patients belonged to the mixed dentition group, 4 (31%) had ORIF, 4 (31%) had soft diet, and 5 (38%) had MMF. Fifteen patients represented the permanent dentition group, and four (27%) had ORIF, eight (53%) had a soft diet, and three (20%) had MMF. Here, significant differences were noticed between the first group (0–5 years) (0%) and the second group (6–11 years) (38%) concerning closed management (*p* = 0.002) and between the first group (0–5 years) (0%) and the third group (12–16) (20%) (*p* = 0.022).

The follow-up ranged from a minimum of 6 to a maximum of 120 months (mean: 22 months).

### 3.5. Hardware Removal

Out of 82 patients treated with ORIF, 15 patients underwent hardware removal (18%). Fourteen patients had a single condylar fracture, ten of which were scheduled and four due to infection (one patient), dental extractions (two patients), or aesthetic reasons (one patient). One patient with a double condylar fracture underwent a scheduled plate removal. In total, 11 out of 15 plate removals were scheduled.

A significant difference was noted between the mixed dentition (81%) and the permanent dentition (97%) groups regarding the rate of plates left in situ (*p* = 0.001).

A significant difference was also noted concerning plate removal after one year; six patients were in the mixed dentition group (11%) and no one was in the permanent dentition group (*p* = 0.001).

### 3.6. Complications

During the first year of follow-up, of the 21 children from 0 to 5 years of age, 17 had no complications (76%), 1 had an infection, 2 had mild malocclusion, and 1 had a slight deviation on opening.

Of the 55 children from 6 to 11 years, 44 had no complications (80%). Three patients had lower lip hypoesthesia, one patient had an asymmetry, one patient had an infection, in one patient, an osteosynthesis screw was too long in the joint space, and two patients had partial palsy of the zygomatic and buccal branches, one patient had an aseptic necrosis of the fractured condyle, one patient a deviation of mouth opening and one patient had pain in the temporomandibular joint. The patients with lower lip hypoesthesia also had intraoral ORIF for mandibular body or symphysis fractures.

Of the 104 children and adolescents from 12 to 16 years, 74 (71%) patients had no complications. One patient had an asymmetry, ten patients had malocclusion, one patient had hypoesthesia of the lower lip, two patients had pain in the temporomandibular joint, one patient had an infection, three patients had complete frontal branch paralysis, three patients had a slightly limited mouth opening, two patients had a deviation during mouth opening, one patient had buccal branch paresis, three patients had mild frontal branch paresis, one patient had a salivary fistula, one patient had dehiscence, one patient had to undergo a re-do operation. The patient with lower lip hypoesthesia also had intraoral ORIF for mandibular body fracture.

At the last follow-up, 18 (85%) children in the deciduous dentition group were well. One patient had a shorter condyle and deviation on mouth opening, one patient had a minimal deviation on mouth opening, and one patient had a minimal malocclusion. In this group, one patient had asymmetry.

In the mixed dentition group, 49 (89%) patients had no complications. One patient had a deviation on mouth opening and a shorter condyle, one patient had a minimum deviation on mouth opening, one patient had a mild malocclusion. The patient with aseptic necrosis went on to develop trismus, TMJ pain and malocclusion. Another patient had TMJ pain and deviation on mouth opening, and one patient had hypoesthesia of the lower lip. In this group, three patients presented with asymmetry.

In the permanent dentition group, 91 (87%) patients had no complications. One patient had TMJ pain, two patients had frontal branch paresis, five patients had minimal asymmetry and deviation on mouth opening, one patient had restricted mouth opening, one patient had salivary fistula, one patient had minor malocclusion, and two patients had TMJ pain and deviation. In this group, six patients had asymmetry.

Therefore, at the last follow-up visit, the great majority (>85%) of patients were well, with fewer patients affected by malocclusion, functional limitations, and pain and a reduction in the number and severity of the neurologic complications.

No significant difference was observed between the type of management and outcome in terms of the number of complications or lack thereof in the first year (*p* = 0.57) or after one year (*p* = 0.60).

Concerning asymmetry, out of 47 patients who had MMF, 42 (90%) did not show asymmetry, and 5 (10%) did. The 51 patients with expectant management were all symmetric, and out of 82 ORIF patients, 77 (94%) were symmetric, and 5 (6%) were asymmetric. A significant difference was observed between the group with MMF (10%) and the soft diet (0%) concerning asymmetry.

**Figure 3:**
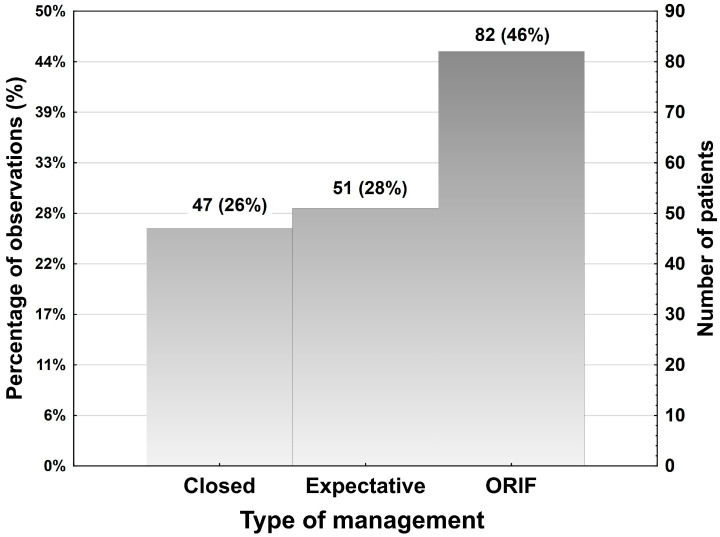
Type of management in the study sample.

A histogram with the three management strategies in the study sample is shown below (Figure 3).

## 4. Discussion

Within our study sample of 424 pediatric patients with mandibular fractures, 42% had one and 9% had two mandibular condyle fractures. This prevalence highlights the high frequency of this fracture localization and is in keeping with previous studies [1,2,3,4,5].

The most common cause of injury in the deciduous dentition group was falls, and in the mixed and permanent dentition groups, RTAs were the most common cause, which is also in keeping with most of the epidemiologic studies [3,4,5].

Pre-school children (<5) are mostly under tight parental supervision, school-age children (6–12) play sports, and adolescents use bicycles, skateboards or scooters as transportation means and for leisure without parental supervision and are more prone to RTAs because of lack of experience and possibly some elements of impulsivity and risk-taking behaviors [16,17].

Administering antibiotics and corticosteroids for periods longer than one week in most patients (94%) was an unexpected finding. Antibiotic prophylaxis in mandibular fractures is indicated for up to 24 h after suture in mandibular fractures that communicate with the oral cavity or skin through a laceration [18,19]. Liberal prolonged use of antibiotics beyond 24 h did not show any advantage in infection rate reduction and is associated with adverse reactions such as allergies, diarrhea, and fatigue [19]. Furthermore, it fosters antimicrobic resistance, blunting antibiotic efficacy globally [19]. Seven-day-long antibiotic prophylaxis appears to be “angst-based” rather than evidence-based, driven by the surgeons’ fear of complications due to contamination of the fracture by the oral biome.

Corticosteroids combat edema and, therefore, reduce postoperative pain, significantly improving the patient’s comfort in the postoperative period but may impair wound healing [20].

Therefore, antibiotic prophylaxis—an essential element in perioperative maxillofacial fracture management—should be used ideally for up to 24 h after suture but not longer [19].

The management strategies (expectant, closed or open) showed marked differences among the participating units.

The above was unsurprising, given the lack of consensus on the topic [21].

Indeed, most authors in English-speaking countries favor a closed or expectant management, based on the notion that the condylar process is a growth center capable of remodeling and, therefore, consider an operation to be unnecessary, risky for the facial nerve and detrimental for mandibular growth due to devascularization of the bone and the additional scarring of surgery [2,5,9,22,23,24,25,26,27]. ORIF is indicated only in cases of condyle dislocation in the middle cranial fossa, the presence of a foreign body, severe malocclusion, or if the dislocated proximal fragment impairs the mouth opening [28]. Incidentally, in the USA, a more conservative approach than in mainland Europe is also applied to the treatment of adult condylar fractures, as highlighted in a survey conducted by Kommers et al. [29]. In the present analysis, the units in India and the UK managed their patients either expectantly or with MMF, no patients underwent open surgery.

On the other hand, some surgeons in mainland Europe favor an operative approach, even in young children and for less restrictive indications than the ones above, based on evidence of suboptimal outcomes after closed treatment and excellent results after ORIF [20,21,22,23,24,25,26,27,28,29,30,31,32]. The units in Slovenia and Austria treated most of their patients with ORIF. Already in the 1990s, maxillofacial surgeons in Austria and Germany around the Alpine region developed a keen interest in treating condylar fractures with open surgery, possibly driven by the prevalence of mandibular fractures due to sports accidents. Therefore, ORIF became the standard treatment for displaced condylar fractures, and techniques were transmitted to younger generations of surgeons.

In the present series, there was just one endoscopic-assisted approach, likely because the technique relies on a different skill set than the ones that are familiar to the maxillofacial surgeon and generally requires longer operating times than external approaches, which makes it a daunting proposition for most trauma surgeons with a busy schedule in this age of time-pressured operations [33]. However, it has the crucial benefits of a reduced risk of facial nerve injury and an intraoral “invisible” scar [15,31,34,35,36,37,38,39].

A bite-raising appliance (hypomochlion) on the molar teeth of the fractured side allows the proximal fragment to straighten itself up [40,41]. Furthermore, active laterotrusion contralateral to the fractured condyle and elastic traction stimulate condylar remodeling in children [42]. The authors of the present study did not use the above functional protocols, possibly due to a lack of knowledge or expertise with these techniques.

The statistical analysis in this sample did not identify significant differences in the management concerning fracture localization. However, there was a tendency to treat condylar base fractures with ORIF, which is in keeping with clinical practice.

Significant differences were identified between the fracture type (non-displaced, displaced or comminuted) and the management (expectant, closed or ORIF), indicating a clear preference for expectant or closed management in non-displaced fractures. On the other hand, most of the displaced fractures were treated with ORIF, reflecting a preference for open surgery in displaced fractures.

The patients’ age did not affect the management choice of 180 patients with single condyle fractures. On the other hand, in the 37 patients with double condyle fractures, none of the children in the deciduous dentition group received MMF, in contrast to the patients in the mixed and permanent dentition groups. This observation is in accordance with clinical practice, as MMF has several drawbacks, especially in children with deciduous or mixed dentition, as discussed below [40,43,44,45].

Concerning condylar head fractures, most centers treated them conservatively. However, the groups in Ljubljana (Slovenia) and Salzburg (Austria) performed ORIF on non-comminuted diacapitular pediatric fractures via a preauricular approach using two positional screws. This approach reflects the tradition, the teaching and the expertise in the above departments [32,46].

If the condyle has reached a sufficient size and the proximal fragment is not comminuted, two positional screws are the ideal solution for osteosynthesis, according to a panel of European experts who met at the International Bone Research Association (IBRA) Symposium in 2012 and as demonstrated in clinical practice [15].

A 3D model of the fractured condyle gives the surgeon a precise impression of the proximal fragment’s size, position and orientation with respect to the distal segment. The surgeon can do a model operation, thereby selecting screws of adequate length and minimizing the risk of revisions [46].

Most of the hardware in ORIF was titanium (95%) and 5% resorbable. This observation mirrors the consensus among surgeons that titanium has better mechanical properties, allowing for the use of implants of smaller dimensions than resorbable hardware for mandibular fractures, which is particularly advantageous in children, who have smaller dimensions than adults [47,48]. Furthermore, resorbable plates are cumbersome, requiring more time and patience [48].

The rate of plate removal was 18% in this pediatric population, per previously published studies [44,48]. In children above 12 years of age, hardware was left in situ in 97% of the patients, like in adults, as per expert advice [15]. Otherwise, in the deciduous and mixed dentition, plate removal was mainly scheduled (11 out of 15), possibly because of concerns regarding teeth buds, growth hindrance and drifting of the plates due to apposition of new bone [48]. Removing plates implies a second hospital admission, general anesthesia, and, in extraoral access, a risk of injury to the facial nerve branches. So far, there is no convincing evidence of growth restriction or plate migration in pediatric mandibular fractures [44,47]. However, screws in condylar heads may exert a stress-shielding effect and cause partial condylar head resorption in up to 30% of the patients [49]. Therefore, some experts routinely remove condylar head screws and recommend hardware removal in this localization [49].

Stress shielding is defined as reducing physiological mechanical demands on the bone, resulting in osteopenia as a functional adaptation.

A separate article from the WORMAT pediatric group will deal specifically with the topic of metalwork removal after maxillofacial trauma in children.

Closed management is synonymous with MMF, and it was used for variable periods from 1 to more than four weeks in our study. It is hard to say if MMF was a proper immobilization or rather in the form of light guiding elastics as a functional treatment, as, regrettably, these two different treatments are often both referred to as MMF. The experts agree that immobilization of the temporomandibular joint is counterproductive as it promotes pain, functional limitations and even ankylosis under certain conditions [25,42,44,50,51,52]. MMF should be avoided, especially in children with deciduous and mixed dentition, as it can cause avulsion in deciduous teeth and damage permanent teeth buds [44]. Indeed, it was never used for children under 5 in the present study. On the contrary, guiding elastics and consistent repetition of protrusions and lateral excursions of the mandible are essential for functional rehabilitation in adults and children and for promoting condyle remodeling, especially in children under 6 [25,41,42,50].

A possible confounding element in the present analysis is that 35 patients also had additional mandibular non-condylar fractures. This fact may explain why a few patients had lower lip hypoesthesia. However, surprisingly, there was no correlation between the number of fractures per patient and the use of MMF.

Overall, 56% of the patients who had ORIF also had MMF. Fracture immobilization is generally sought in case of insufficient fixation because of fear of displacement in the postoperative period as a “salvage” option. Contrarily, prescribing light guiding elastics after open surgery is part of the neuro-muscular rehabilitation process after stable osteosynthesis. Regrettably, it is impossible to know more in detail if strict immobilization or guiding elastics were used in the present population. Children poorly endure strict MMF, which implies anxiety, airway restriction, decreased nutrition and insufficient hygiene [40,44,45,47]. Bearing the above in mind and considering the quicker healing in children, surgeons should strive to abolish jaw immobilization in these groups of patients [47].

Every protocol had a low rate of asymmetry, and the 51 patients with expectant management were all symmetric. The fractures treated with MMF were mostly non-displaced, so there was an obvious selection bias.

Unsurprisingly, different units favored different protocols: this is traceable to the country’s teaching, tradition, and the department’s expertise.

The most important finding of the present analysis was that no significant difference was observed between the three types of management and their outcomes in terms of the number of complications or lack thereof.

Therefore, if the indication and treatment are correct, the above three management strategies are adequate. This choice requires adequate know-how.

This study has several limitations: A fourth management option for pediatric mandibular condyle fractures is represented by functional protocols. Functional protocols were not applied in our sample, possibly because the present study’s authors have a surgical and not an orthodontic background. Further limitations are the following: the confounding factor that 35 patients also had additional mandibular non-condylar fractures, the retrospective design with inevitable information loss, the lack of a standardized, validated outcome evaluation such as the Helkimo Anamnesis and Dysfunction Index, and the absence of the category “dislocated” among the fracture descriptors, the short follow-up for many patients [53]. 

Clinical asymmetry is evident only in gross asymmetry, and in the absence of radiologic measurements, it may have been underreported. The study’s strength is the multicentric global contribution, which displayed three different approaches and enabled the reach of a much larger sample than in a single institution.

The literature search was extended to the German language and included papers from several continents to reflect different perspectives.

## 5. Conclusions

The key finding of the present study was that expectative management, MMF, and ORIF were all effective in treating pediatric mandibular condyle fractures, with a low incidence of complications.

In fractures without displacement or minimally displaced, expectant treatment was highly successful thanks to the remarkable healing capacity of the condylar region in children. Fractures with displacement were managed in various ways, depending on the local tradition and expertise. It is worth highlighting that true jaw (and joint) immobilization should be foregone in pediatric patients because of its drawbacks. On the contrary, it appears from the literature that active mandibular movements and light guiding elastics improve outcomes in cases of dislocation, promoting functional rehabilitation and condylar remodeling in children [41,42].

In the present analysis, for displaced fractures, even in children with deciduous and mixed dentition, an expert open approach could help restore the integrity, position and function of the hard and soft tissues without negative repercussions on mandibular growth, in agreement with the literature [25,32,54,55]. Facial nerve injuries were rare and mostly temporary [32,56]. This surgery is challenging and has high stakes and must be carried out with the least possible additional trauma.

The practical implications of the present analysis are as follows: 1. Consider implementing a functional protocol for treating displaced mandibular condyle fractures in children, which may involve collaboration with an orthodontist. 2. Use shorter courses of postoperative antibiotics in open reduction and internal fixation (ORIF) procedures. 3. Explore the use of endoscopic approaches for condylar base fractures. 4. It is possible to operate on growing condyles without arresting condylar development if the operator has adequate training and expertise.

## Figures and Tables

**Table 1:** Maxillofacial surgery units participating in the World of Oral and Maxillofacial Trauma collaboration for studying pediatric maxillofacial trauma listed in alphabetic order.

Country	City	Institution
Austria	Salzburg	Dpt. Oral and Maxillofacial Surgery, Paracelsus Medical University
Belgium	Leuven	Dpt. Oral and Maxillofacial Surgery, University Hospitals Leuven
Bosnia and Herzegovina	Mostar	Clinic for ENT and OMS University Clinical Hospital
Brazil	São Paulo	Dpt. Diagnosis and Surgery, Araraquara Dental School—UNESP—State University
Bulgaria	Plovdiv	Dpt. Maxillofacial Surgery, Medical University
Croatia	Zagreb	Dpt. Maxillofacial Surgery, University Hospital Dubrava
India	Aligarh	Dpt. Oral and Maxillofacial Surgery, Aligarh Muslim University
Iran	Mashhad	Dpt. Oral and Maxillofacial Surgery, Mashhad Dental School, University of Medical Sciences
Italy	Turin	Division of Maxillofacial Surgery, Città della Salute e della Scienza, University of Turin
Nigeria	Ibadan	Dpt. Oral and Maxillofacial Surgery, College of Medicine, University of Ibadan
Serbia	Belgrade	Clinic of Maxillofacial Surgery, School of Dental Medicine, University of Belgrade
Slovenia	Ljubljana	Dpt. Maxillofacial and Oral Surgery, University Medical Centre
Tanzania	Muhimbili	Dpt. Oral and Maxillofacial Surgery, University of Health and Allied Sciences
United Kingdom	Dundee	Dpt. Oral and Maxillofacial Surgery, University of Dundee

**Table 2:** Type of management and number of patients treated in each unit. (ORIF = open reduction and internal fixation).

	Management			
Country	Closed	Expectative	ORIF	Number of Patients
ITALY	1	7	2	10
Percentage (%)	10%	70%	20%	
BULGARIA	12	0	0	12
Percentage (%)	100%	0%	0%	
SLOVENIA	0	11	40	51
Percentage (%)	0%	22%	78%	
BELGIUM	6	0	0	6
Percentage (%)	100%	0%	0%	
AUSTRIA	0	11	17	28
Percentage (%)	0%	39%	61%	
CROATIA	7	4	5	16
Percentage (%)	44%	25%	31%	
SERBIA	9	2	0	11
Percentage (%)	82%	18%	0%	
INDIA	8	2	0	10
Percentage (%)	80%	20%	0%	
BOSNIA	0	2	1	3
Percentage (%)	0%	67%	33%	
BRAZIL	0	2	7	9
Percentage (%)	0%	22%	78%	
IRAN	2	0	10	12
Percentage (%)	17%	0%	83%	
TANZANIA	1	3	0	4
Percentage (%)	25%	75%	0%	
UK	1	6	0	7
Percentage (%)	14%	86%	0%	
NIGERIA	0	1	0	1
Percentage (%)	0%	100%	0%	
**Number of patients**	**47**	**51**	**82**	**180**

## Data Availability

Additional data are available upon request to the corresponding author.
